# Des métastases osseuses d’un mélanome malin détectées sur une TEP au 18F-FDG avec scintigraphie du squelette normale

**DOI:** 10.11604/pamj.2017.27.209.11009

**Published:** 2017-07-20

**Authors:** Abderrahim Doudouh, Abdelhamid Biyi

**Affiliations:** 1 Department of Nuclear Medicine, Mohammed V Military Teaching Hospital, Mohammed V University of Rabat, Morocco

**Keywords:** Scintigraphie osseuse, mélanome malin, TEP au 18-FDG, métastases osseuses, Bone scintigraphy, malignant melanoma, 18-FDG PET, bone metastases

## Image en médecine

Nous rapportons le cas d’une patiente âgée de 30 ans, opérée deux ans plus tôt pour un mélanome de la fesse droite et qui a consulté pour les douleurs rachidiennes persistantes. La scintigraphie osseuse (SO) était tout à fait normale (A). Compte tenu de l’histoire médicale de la patiente, une tomographie par émission de positons (TEP) au 18-FDG a été pratiquée dans les 30 jours qui ont suivi et a montré de multiples foyers d’hypermétabolisme aux niveaux de la fesse droite, d’adénopathies inguinales du même coté, du poumon gauche et de l’ensemble du squelette (B). La patiente est malheureusement décédée dans les jours qui ont suivi avant toute tentative de traitement. Les métastases osseuses des mélanomes malins sont rares. Quand elles surviennent, elles sont typiquement ostéolytiques dans 87,5% des cas avec une infiltration médullaire dans 91,6% des cas. Pour cette dernière raison, la pratique d’une TEP au 18-FDG s’avère très contributive. Notre cas illustre clairement sa supériorité de la TEP au 18-FDG par rapport à la SO dans cette indication. Actuellement et selon les données de la littérature, l’inscription d’une TEP au 18-FDG dans le cadre du bilan d’extension ou bien de surveillance d’un mélanome malin s’avère largement justifiée.

**Figure 1:**
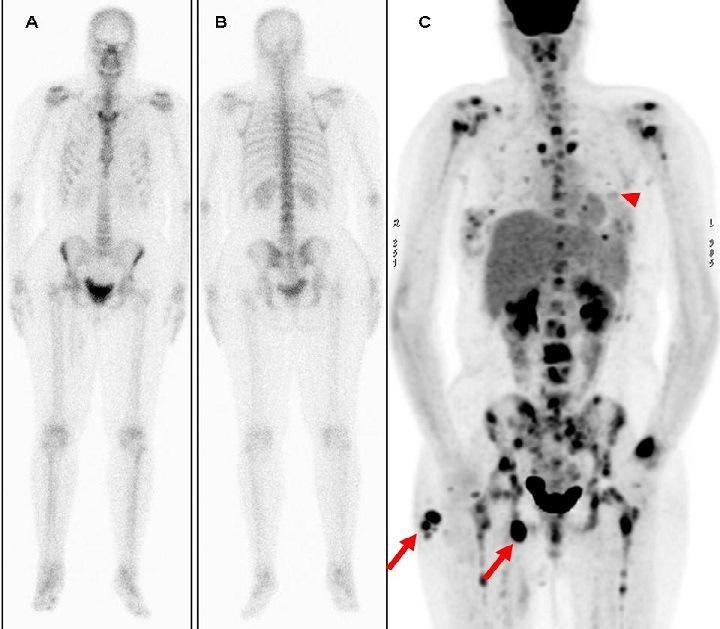
Scintigraphie osseuse en vue antérieure (A) et postérieure (B) montrant une fixation normale sur l’ensemble du squelette. Aucun foyer de localisation secondaire n’a été décelé. (C) TEP au 18-FDG montrant de multiples foyers d’hypermétabolisme aux niveaux de la fesse (flèche) et d’adénopathies inguinales droites (flèche), du poumon gauche (pointe de flèche) et de l’ensemble du squelette

